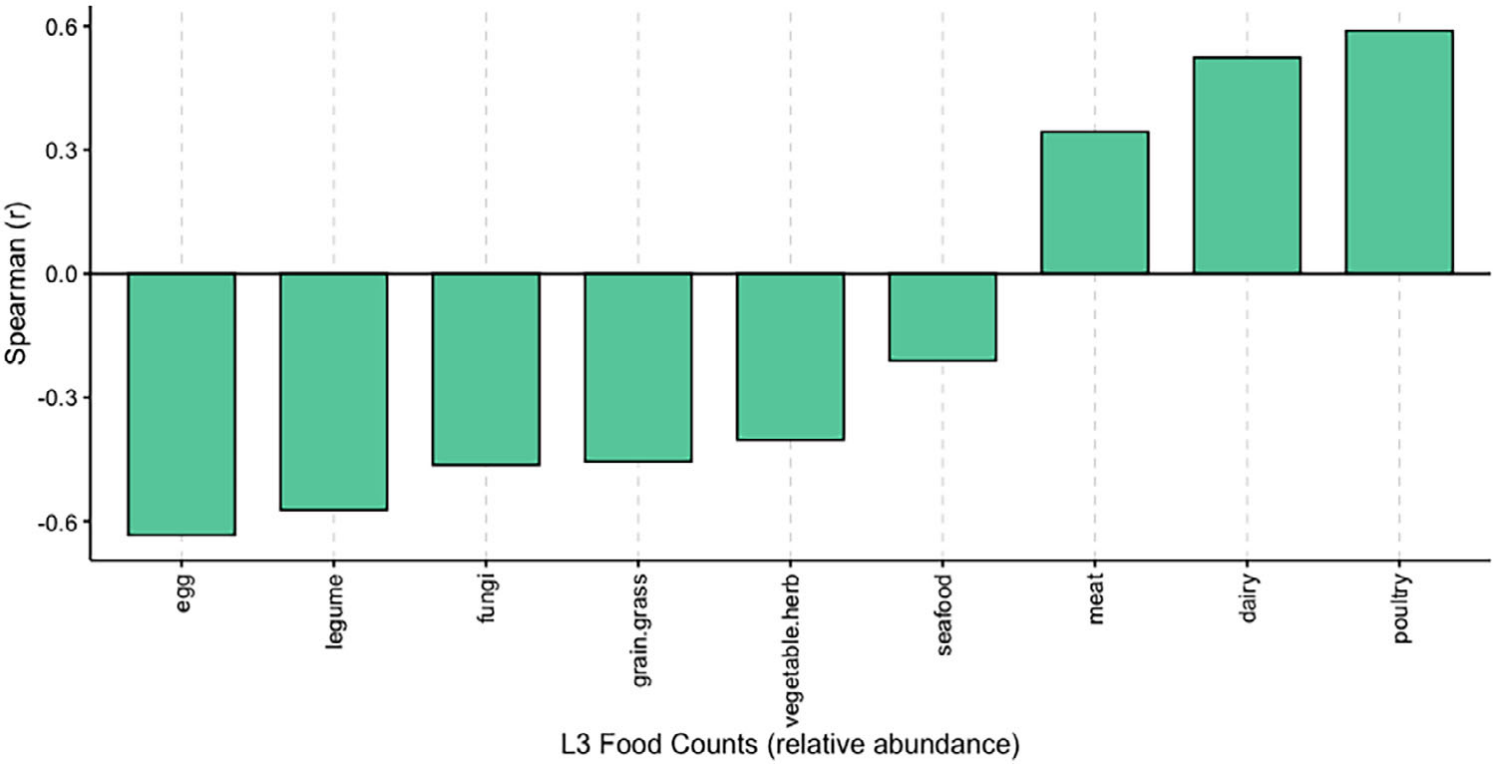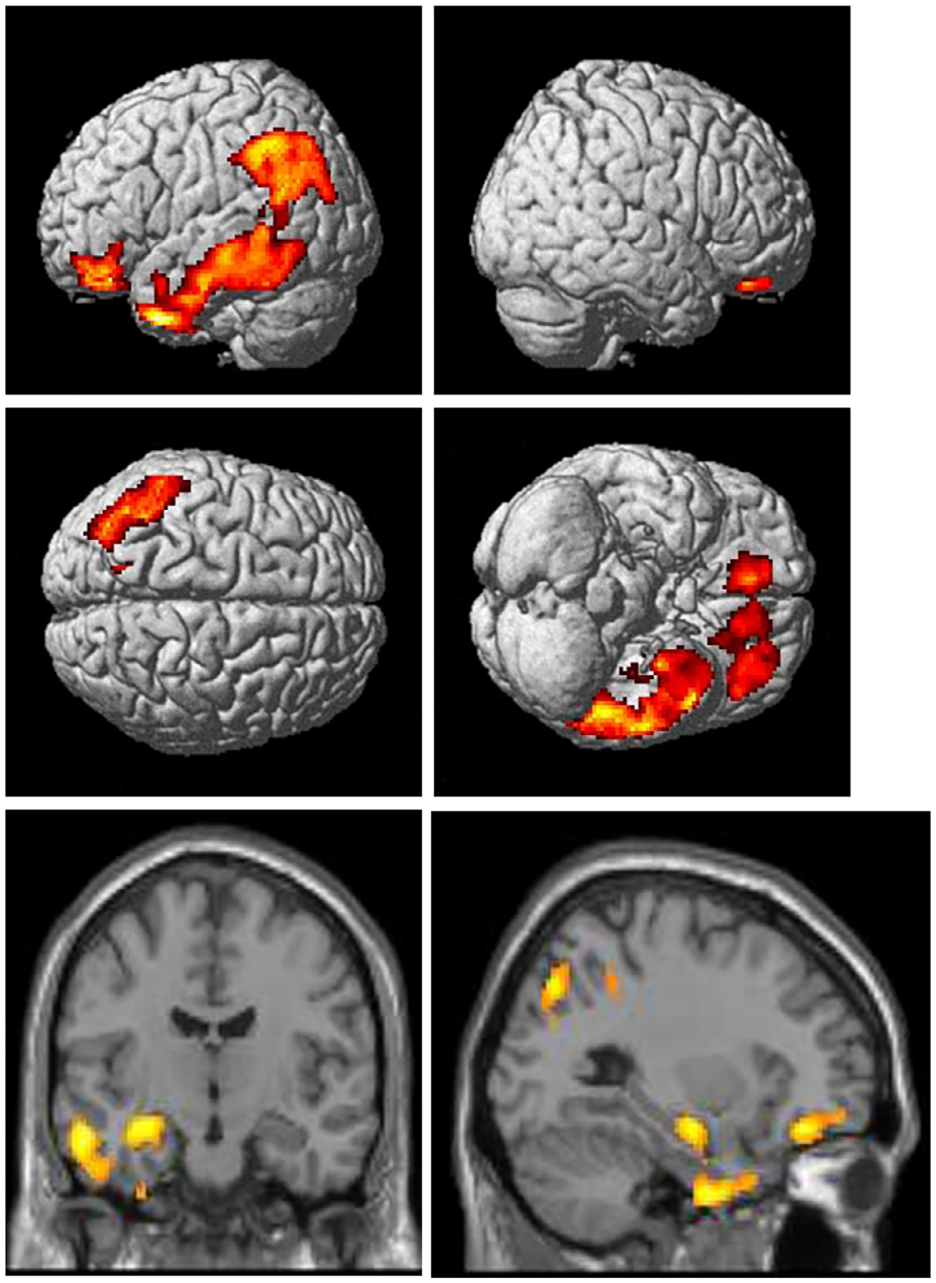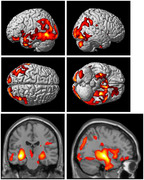# Foodomics: Links to cognition and AD markers of disease

**DOI:** 10.1002/alz.089889

**Published:** 2025-01-09

**Authors:** Jennifer S Labus, Kwangsik Nho, Simone Zuffa, Richa Batra, Michael Meehan, Kiana West, Jasmine Zemlin, Andres Mauricio Caraballo‐Rodriguez, Pieter Dorrestein, Rima Kaddurah‐Daouk, Alzheimer's Gut Microbiome Project Consortium (AGMP)

**Affiliations:** ^1^ UCLA Vatche and Tamar Manoukian Division of Digestive Diseases, Los Angeles, CA USA; ^2^ Indiana Alzheimer Disease Research Center, Indianapolis, IN USA; ^3^ University of San Diego, San Diego, CA USA; ^4^ Institute for Computational Biomedicine, Englander Institute for Precision Medicine, Department of Physiology and Biophysics, Weill Cornell Medicine, New York, NY USA; ^5^ University of California, San Diego, La Jolla, CA USA; ^6^ University of California San Diego, San Diego, CA USA; ^7^ Department of Psychiatry and Behavioral Sciences, Duke University, Durham, NC USA; ^8^ Duke University, Durham, NC USA

## Abstract

**Background:**

Diet has been associated with memory, emotion/stress regulation, structure and function of the hippocampus and amygdala and attenuation of cognitive aging. There is a well‐recognized lack of reliability in self‐reported dietary intake and great interest in objective metabolic readout of dietary patterns. In this study we constructed dietary profiles from untargeted metabolomics data using a novel metadata‐based source annotation method developed at the Dorrestein Lab, also referred to as “foodomics”. The aim of was to link these objective metabolic readouts of dietary intake to cognition and AD markers of disease.

**Method:**

Untargeted metabolomic profiling (LC‐MS/MS) was applied to blood samples from 872 individuals from the ADNI cohort (182 cognitive normal individuals, 449 MCI, 103 significant memory concern, and 139 AD); for whom brain imaging data was also collected. Principal component analysis (PCA) was to derive a dietary profile that explained the most variance in the dataset. Whole‐brain voxel‐wise analysis was applied to determine association between dietary intake profiles and brain amyloid‐β deposition (amyloid PET), gray matter density (brain atrophy; MRI), and glucose metabolism (FDG PET) using a multivariate regression analysis with SPM12. The general linear model was used to examining the associations of the dietary profile with cognition and AD markers. Covariates included age, education, sex, and BMI.

**Result:**

Correlation between the first principal component (PC1) and the relative abundances of foods at level 3 of the food ontology showed that PC1 explained variance derived from food intake within poultry, dairy, meat, egg, vegetable, legume and seafood categories (Figure 1). Greater intake of poultry, dairy, and meat was significantly associated with 1) reduced glucose metabolism and brain gray matter atrophy in the frontal, parietal, and temporal (including the hippocampus; Figure 2,3) and 2) difficulties with executive functioning (p=0.03) and memory at baseline (p=0.002), longitudinal decreases in memory functioning over time (p=.02), and increases in baseline plasma levels of neurofilament light chain at baseline (p=.02).

**Conclusion:**

Dietary intake selectively influences glucose metabolism and gray matter atrophy of brain regions preferentially targeted in AD, cognition and neurofilament light chain. This proof‐of‐concept study supports the hypotheses that diet influences the progression of the disease. NIA U19 AG063744